# Numerical and Field Measurement Investigation on Foundation Pit Excavation Blasting of Anchor in Suspension Bridge

**DOI:** 10.3390/s22228952

**Published:** 2022-11-18

**Authors:** Lei Yan, Xiaoying Gou, Zengshun Chen, Yunfei Fu, Guo Li, Longfei Cheng, Xuanyi Xue, Yu Jiang

**Affiliations:** 1 School of Civil Engineering, Chongqing Three Gorges University, Chongqing 404100, China; 2 State Key Laboratory of Mountain Bridge and Tunnel Engineering, Chongqing Jiaotong University, Chongqing 400074, China; 3 School of Civil Engineering, Chongqing University, Chongqing 400045, China; 4 Department of Civil and Environmental Engineering, The Hong Kong University of Science and Technology, Clear Water Bay, Kowloon, Hong Kong 999077, China

**Keywords:** suspension bridge, foundation pit, blasting excavation, risk control measures

## Abstract

The foundation pit of a suspension bridge project in the Three Gorges Reservoir area is investigated in this paper. The pit is located under an unstable rock mass and landslide body; its base lithology is mudstone. The bridge foundation pit project mainly adopts blasting excavation to accelerate construction progress. However, as a hazardous technique to engineering safety, the explosion vibration easily causes deterioration of the surrounding strata, thereby inducing slope instability and rock mass collapse. Besides, three major challenges should be considered: complex terrain conditions, difficulties in the blasting excavation of anchors, and the extremely high risk of construction. Therefore, comprehensive risk control measures using the methods of hierarchical excavation and minimum charge blasting are put forward. After the measures were verified to be feasible through finite element simulation, it was successfully applied to actual construction. In addition, this paper proposes using fiber concrete to reinforce slope retaining walls, and simulates the reinforced effect based on the research above. The results indicate that the risk control scheme is reasonable, which not only ensures the construction process but also guarantees the stability of the slope and unstable rock body. At the same time, the slope is reinforced with fiber concrete, which effectively decreases the protection wall thickness. Finally, the article can provide a valuable reference for similar engineering projects around the world.

## 1. Introduction

Currently, a slope protection wall is usually used, which is poured using conventional Portland cement concrete. The construction safety risk is greatly increased when blasting excavation must be carried out for an engineering project, where it takes place in certain types of terrain, such as a wide, unstable rock belt. However, conventional preventive measures are faced with a series of challenges, such as difficulties in construction, long project periods, high budget expenses and low prediction accuracy. In this regard, many scholars have tried to introduce interdisciplinary approaches to eliminate intractable engineering issues and have made effective progress. For example, by introducing new materials, the dangerous area is accurately set up, and the structural performance is also improved with regard to dangerous area reinforcement [1]. Second, an early warning system with a machine learning algorithm or other computer-aided techniques can be applied. Chen Z et al. [2] have made an early attempt in this field. They decomposed the original measurement signal using the ensemble empirical mode decomposition (EEMD) method, and further combined deep neural networks (DNNs), gated recurrent units (GRUs), and long short-term memory networks (LSTMs) to predict subsequent decomposed original measurement signal data. Their technique significantly improves the effectiveness and accuracy of evaluating the dynamic response of structures. Huang et al. [3] also proposed a safety monitoring algorithm and simplified the evaluation of foundation pit deformation by using big data. The results showed that security monitoring by introducing computational assistance technology has a positive effect on the engineering project. Many similar cases can be found in the references. The importance of computer-aided technology was clearly emphasized. With its help, an early warning system can be established, and loss detection can be obtained before or after construction [4]. Therefore, risks can be found in time or even curbed in an engineering project. Moreover, a further set of research was carried out. Puzhen An et al. [5] conducted comparative studies on deep foundation pit construction. Zhang et al. [6] carried out a risk assessment study of foundation pit construction. Li Lin et al. [7] evaluated the impact of foundation pit excavation on adjacent buildings and structures. All results proved that selecting appropriate construction technology is the key to reducing risks. Bin Chen et al. [8], according to the weight-in-motion (WIM) data, established a fatigue load model that reflects the actual traffic conditions of the bridges. Yangjian Xiao et al. [9] came up with two methods based on the strain equivalent principle and Sidiroff energy equivalent principle, respectively, and proposed a means to determine the plastic-damage factor of the concrete’s uniaxial constitutive relation in a specification. Yong-Sheng Yao et al. [10] and Zeng-Shun Chen et al. [11,12] used different measurement systems to achieve good results in agriculture and engineering. Jianmin Hua et al. [13] point out that the BSB (bimetallic steel bar) has a broad application prospect in RC (reinforced concrete) structures in a corrosive environment. Liu Hanlong et al. [14] used microbial geotechnical technology to achieve environmental purification and soil remediation, which is also a new idea for slope reinforcement. The research ideas proposed by the above scholars are worthy of study and reference.

A suspension bridge is the best way to cross deep and wide rivers [15]. Anchorage plays a crucial role in the stability of suspension bridges, and it is the core component to support the main cable, as well as to ensure the stability of the whole main structure of the bridge [16]. Although anchorage is widely used in suspension bridges, it always causes security hazards. Therefore, effectively protecting and eliminating these potential safety risks is a key point in engineering projects. In conventional bridge engineering projects, blasting excavation and mechanical excavation are often used in anchoring foundation pit construction. As an economical and efficient rock mass excavation method, blasting excavation is one of the main means of gravity anchorage foundation pit excavation at present. However, the shock wave and high-pressure gas generated by the explosion will inevitably cause damage to the retained rock mass [17]. Therefore, it is necessary to evaluate the potential risk, and reasonably choose whether to adopt blasting excavation, based on the actual project. For example, the Luding Dadu River Xingkang Suspension Bridge, with gravity anchoring foundation pits, was suitable for blasting excavations. This is because of its gently sloping anchoring site, good geological structure, and lithology. Therefore, only minor and stable damage would be caused to the anchoring foundation pit wall and surrounding rock after blasting excavation.

The anchorage foundation pit of the current work is located under a dangerous rock zone and landslide deposit body; the base rock lithology is mudstone, which is not suitable for blasting excavation in practice. However, in order to accelerate the construction progress, blasting excavation had to be adopted. In this case, blasting vibration is likely to cause the deterioration of surrounding strata [18], inducing slope instability [19] and dangerous rock collapse [20]. Thus, avoiding a series of safety hazards is the key issue of the project. In view of similar engineering situations, scholars have conducted some exploration studies. For example, Zhang Hui et al. [21], adopted the method of anchor bolt support and net jetting C20 concrete to protect the slope in the Qipanzhou Yangtze River Highway Bridge. They aimed at resolving the technical difficulties of the excavation of a gravity rock-socketed anchorage deep-foundation pit in complex terrain. Although they set displacement monitoring points at crucial positions of the foundation pit, there was still uncertainty in determining the key points of the foundation pit, due to the lack of pre-construction scheme simulation. Yang Jichao et al. [22] considered the actual engineering situation of the Qingshuihe Bridge, and further proposed the safety control of the gravity anchorage deep foundation pit of the suspension bridge utilizing layered excavation, bolt hanging net injection, and prestressed anchor cable protection. Although the proposed method effectively prevents the collapse of rock debris in the foundation pit slope, it does not include risk monitoring of the whole safety control process. Results show that there are deficiencies if we only rely on construction personnel to inspect hidden dangers. Xiao Anbin et al. [23] used FLAC3D to conduct a finite element simulation of the pre-construction scheme of the deep foundation pit slope project of the north bank of the Baiyang Yangtze River Highway Bridge. The numerical model established by the finite difference program can obtain the dynamic deformation and stability evolution law of the rock mass of the deep foundation pit slope project, which is conducive to the construction and the determination of the key safety points. However, the limitation was that it only used the station to monitor the displacement in the field, which lagged behind the judgment of the stability of the slope.

To sum up, many scholars have solved the problem of slope instability in the excavation process by taking preventive measures before construction, or the timely detection of safety risks through monitoring during construction. However, few have introduced the methods of other disciplines, using both the finite element simulation of the prevention program, and the real-time monitoring of the construction process of the program to conduct a dynamic stability analysis of the slope [24], and even to eliminate the safety hazards.

**Figure 1:**
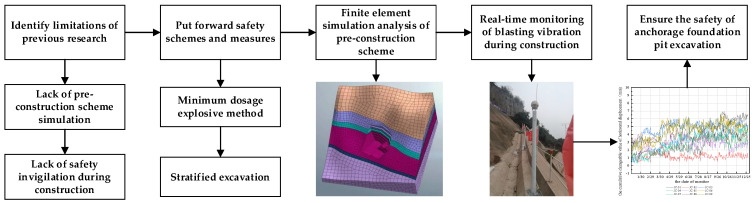
Flow Chart of Research Methods.

The anchorage foundation pit of the suspension bridge is currently facing a series of technical difficulties, such as the consideration of special terrain conditions, blasting difficulties of anchorage excavation, and high risks in construction. Therefore, how to carry out effective risk control is of great significance. Based on the studies of construction challenges and detailed risk control measures, this paper puts forward several comprehensive risk control measures using the method of hierarchical excavation and minimum charge blasting. In addition, the paper verifies the feasibility and influence of the measures, and summarizes their advantages and disadvantages, which can further provide a reference for similar engineering projects around the world. The methodology of this study flow of the full text is shown in Figure 1.

## 2. Risk Control Measures

**Figure 2:**
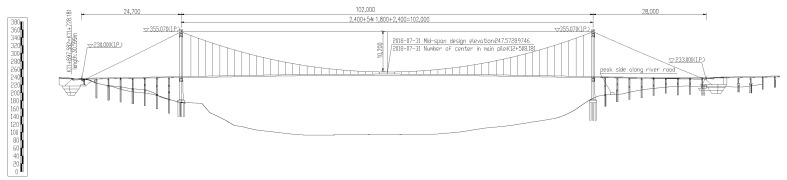
Elevation of suspension bridge.

**Figure 3:**
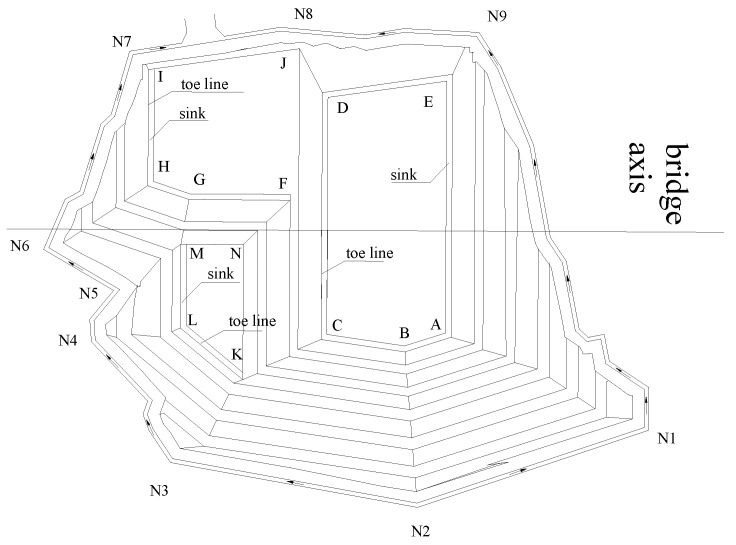
The plan of the anchorage foundation.

The proposed project is the suspension bridge anchor foundation pit. One side is adjacent to the high and steep slope, and the other side is adjacent to the Yangtze River. Its elevation is shown in Figure 2. The suspension bridge in the project uses gravity anchors on both banks. The anchorage area is located in the mid-slope, and the altitude of the ground surface distributes between 210 m and 250 m. The designed height for the foundation pit bottom is 198 m (from the bottom of the anchor blocks). In addition, the area of anchorage foundation pit excavation is 5958 m^2^, the area on the bottom of the foundation pit is 1026 m^2^, and the slope area is 5633 m^2^. Therefore, the total volume of the anchorage foundation pit excavation is 110,400 m^3^. Futhermore, the basement of the anchorage foundation pit is located on a moderately weathered mudstone stratum, and its allowable bearing capacity is not less than 1.35 MPa. After excavating, the height of rock slopes are between 12.0 m and 52.0 m, mainly consisting of mudstone and sandstone, covered with a small amount of rocky soil. The maximum grade of the foundation pit slope is 1:0.3, while the minimum is 1:1, and the highest level for the slope is 6. The schematic design of the foundation pit excavation is shown in Figure 3.

The bridge sits in complex geographical conditions. The cable support tower base is located not only in a cliff area, but also across the riverside highway. Therefore, there are five main engineering challenges, as follows:

(1) Challenges for the oversized anchorage foundation pit area and excessive excavation volume;
(2) A dangerous rock zone is above the construction area of the anchorage foundation pit. Also, solitary boulders are observed above the landslide body. Therefore, serious risks exist in the construction;
(3) Vibration-induced influence on the slope stability due to the blasting excavation cannot be ignored;
(4) Significant elevation differences on the ground surface lead to difficulties in selecting the channel during excavation;
(5) The anchorage site has a large grade of terrain slope and a deep foundation pit.

According to the above technical challenges, a series of comprehensive risk control measures using layered and graded excavation and minimum charge blasting methods are put forward, to meet the safety requirements of the actual engineering project.

### 2.1. Layered and Graded Excavation

**Figure 4:**
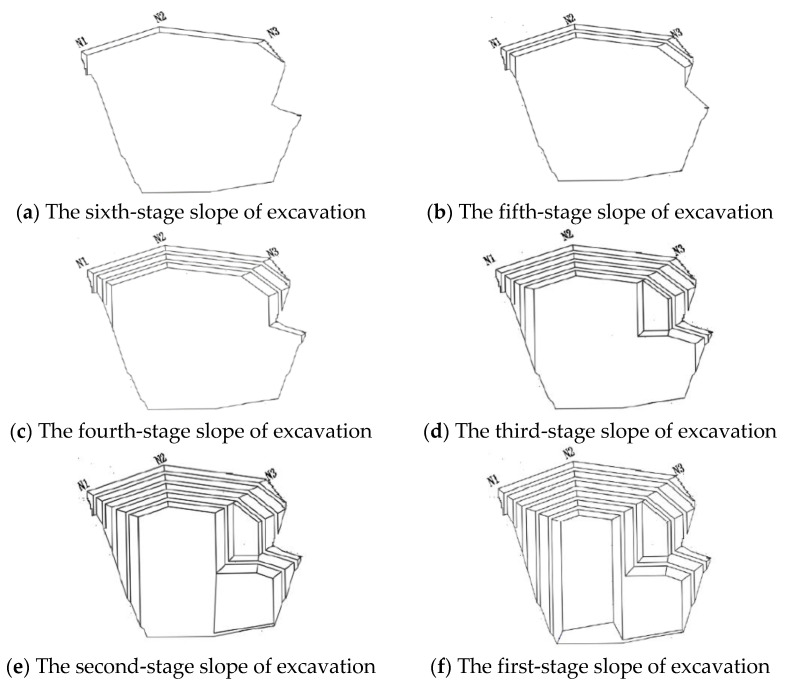
The sequence of the designed excavation.

**Figure 5:**
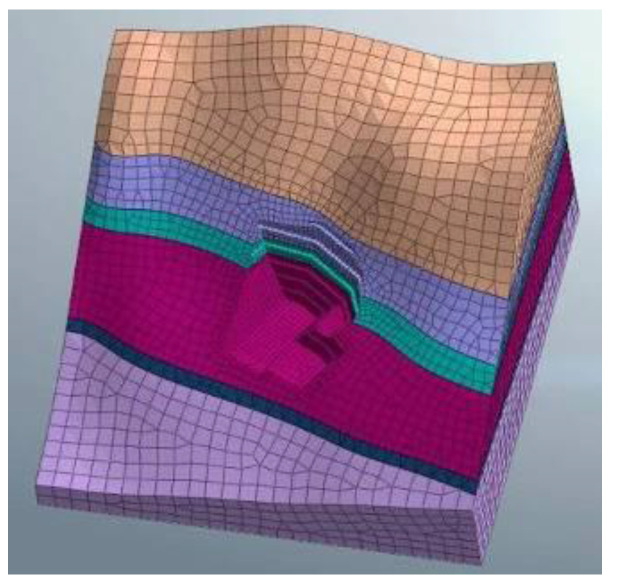
The meshing of surrounding rock.

The vertical slope of the anchorage foundation pit is divided into six layers to control the risks caused by difficulties such as the excessive area and volume of the excavation. Meanwhile, the top–bottom technique is applied for earth rock excavation according to the ground elevation. Each main layer of the slope is cut into multi-thickness pieces based on various geological conditions for easy excavation, and the control value for the maximum excavation depth is limited to 3 m. The sequence of the designed excavation is shown in Figure 3 and Figure 4. The different colors (purple to yellowish brown) from bottom to top in Figure 5 represent the stratified excavation, corresponding to layers 1–6.

It is essential to use some method to protect all layers and slopes, such as anchorage-shotcrete support, bolt-faced walls, or anchor wire-faced walls. The height of rock slope is between 12.0 m and 52.0 m, mainly consisting of mudstone and sandstone, covered with a small amount of rocky soil. The maximum grade of the foundation pit slope is 1:0.3, while the minimum ratio is 1; the highest level for the slope is 6, and the lowest level is 1, respectively. Furthermore, a hand of height slope separately is 8 m and 10 m.

### 2.2. Minimum Charge Blasting Method

Blasting excavation was necessary for the foundation pit due to the high-strength rock body. Therefore, the minimum charge blasting method was conducted for the excavation volume, to reduce the blasting influence on the stability of the slope and unstable rock zone. The minimum dosage explosive method is a method to obtain the total amount of blasting charge by calculating the required blasting load and the maximum amount of blasting charge in a single section. The total amount of blasting charge required by the project is then determined according to the relevant specifications of the excavation of the foundation pit. Thus, the amount of blasting excavation charge will have the least influence on the stability of the foundation pit, that is, the minimum amount of blasting charge required for the excavation project.

## 3. Simulation of Blasting Excavation

### 3.1. Establishment of the Finite Element Model

In the project, conventional mechanical excavation was combined with drilling and blasting techniques for the anchorage foundation pit excavation. The software MADIS/GTS (Geotechnical & Tunnel Analysis System) was applied to conduct the finite element analysis. Firstly, the Mohr–Coulomb elastoplastic material model [25] was used for a direct integral solution in the geotechnical calculation, and the relevant parameters are shown in Table 1. Secondly, in model building, the soil mass was discretized using high-precision 10-node tetrahedral elements, and the grid around the structural elements was encrypted to obtain better convergence and calculation accuracy. The wall protection was simulated by a plate element, and the anchor cable support structure was simulated by an implantable truss and beam element. The size range of the model was X[−130, 130], Y[−130, 130], Z[−70, 120], which was divided into 32,463 nodes and 57,080 elements. Moreover, a linear time history analysis was applied, and the initial geo-stress equilibrium of the analysis model only considered the dead weight stress and ignored the influence of tectonic stress. Since there is no rock mass parameter under saturated water and no slope wetting line under rainstorm conditions in the geological prospecting report, the effects of groundwater and rainfall were not considered in the slope blasting excavation. Finally, the dynamic absorption boundary was adopted in this model, and the viscous boundary was set to consider the influence of energy loss caused by wave energy escape on the dynamic properties of the surrounding rock. The bottom boundary of the model limits the horizontal and vertical displacements of elements, while the side boundary limits the horizontal displacements. A combination of contact element and collision element was used to connect different soil layers and model components. The blasting vibration loading was carried out using the reaction spectrum method. The seven physical parameters of natural weight, saturated compressive strength, Poisson’s ratio, saturated compressive strength, shear angle, internal friction angle and elastic modulus of the three rock strata soil samples were obtained through geological exploration data and indoor physical experiments, as shown in Table 1. However, it was necessary to further calculate the relevant parameters of the Mohr–Coulomb constitutive model adopted in the finite element simulation. In this regard, referring to the calculation method of concrete Mohr–Coulomb parameters by Selimir, the parameters required by five constitutive models, namely: cohesion, internal friction angle, volume modulus, shear modulus, and tensile strength were determined according to the soil samples of the three strata.

The model size range was X [−130, 130], Y [−130, 130], Z [−70, 120]. The soil layers were established, and were divided into 32,463 joints and 57,080 elements. The constraint location was fixed in the bottom area and the other areas were free. The following hypothesis was proposed in terms of modeling: (1) The initial ground stress balance merely considers self-weight stress, but ignores tectonic stress; (2) Current slope blasting excavation overlooked the effect of groundwater and rainfall, due to the lack of rock parameters under saturated moisture conditions, and the slope’s phreatic lines as a result of rainstorms. The overall model is shown in Figure 5. The different colors (purple to yellowish brown) from bottom to top in Figure 5 represent the stratified excavation, corresponding to layers 1–6. The sequence of the designed excavation pit is shown in Figure 4. Figure 4 is composed of six figures (a) to (f), representing the layer-by-layer excavation design plan of the foundation pit, from the sixth level (a) to the first level (f), respectively.

### 3.2. Realization Method for Key Parameters

#### 3.2.1. The Design Parameters of the Supporting Structure

**Figure 6:**
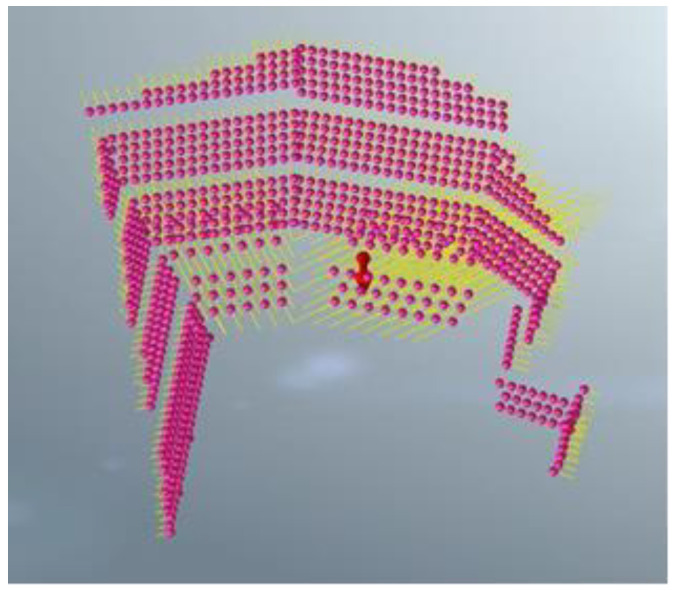
Anchor bar and anchor cable of the foundation pit system.

**Figure 7:**
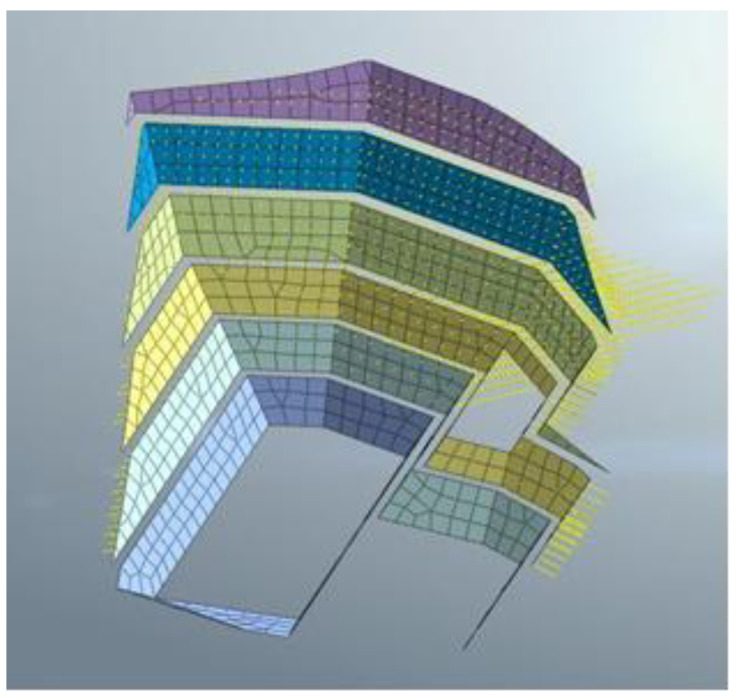
Retaining walls of the foundation pit.

Table 2 lists the design parameters of the supporting structure. Figure 6 shows the anchor bar and anchor cable of the foundation pit system. Figure 7 illustrates the retaining walls of the foundation pit.

#### 3.2.2. Blasting Load

(1) Calculation of the equivalent explosive diameter for the maximum single-blow blasting charge

The calculation of the explosive diameter was performed as follows: the total blasting charge is *Q_ma__x_* = 18 kg, the total length is *L*2 = 1.5 m, the blasting density is *ρ* = 1000 kg/m^3^, the maximum single-blow blasting charge is 18 ÷ 5 = 3.6 kg; the equal explosive diameter is *D* = 2 × (3.6 ÷ 1000 ÷ π ÷ 1.5)0.5 = 0.055 m = 55 mm, and the explosive diameter is approximately 55 mm. In practice, three 32 mm charges were lashed horizontally and placed into a borehole with a cross-sectional area consistent with the equal explosive diameter of 55 mm. 

(2) Blast loading

The blast loading can be simplified into a triangular load curve based on the explosive vibration theory [26]. The performance indices of 2 rock emulsion explosives are shown in Table 3.

The applicable calculation method on peak blasting value is as follows.

(1) Detonation pressure

The calculation of the coupled charging condition is shown as Equation (1).

(1) $$P_{e}=\frac{P_{0}D_{0}{}^{2}}{4}$$

The equation of the uncoupled charging condition is shown as Equation (2).

(2) $$P_{e}=\frac{P_{0}D_{0}{}^{2}}{4}\times \frac{(d_{b}/d_{c}{)}^{6}}{2}$$

*P*_0_—explosive density (kg/m^3^); *D_0_*—explosive detonation velocity (m/s), *d*_b_—explosive diameter (m); *d*_c_—borehole diameter (m).

(2) The initial pressure peaks of the shock wave

The initial pressure peaks of the detonation wave on the borehole wall under the influence of a single borehole are shown in Equation (3).

(3) $$P_{r}=\frac{2\rho_{r}C_{er}}{\rho_{r}C_{er}+\rho_{0}D_{0}}\cdot P_{e}$$

$P_{r}$—the initial pressure peaks of the shock wave in rock body (Pa), $\rho_{r}$—density of rock body (kg/m^3^); $C_{er}$—compression wave velocity of the rock body (m/s).

Equation (3) indicates that pressure peaks on the borehole wall differ when the same explosive explodes in various rocks; $P_{r}$ is enlarged with increasing wave impedance in rock ($\rho_{r}C_{er}$). The pressure increases with the enhancement of surrounding rock (smaller level) and vice versa. However, the wave transmission process between the borehole wall and bench slope wall follows the wave attenuation law, and the pressure values of the two are not equal.

(3) Blasting equivalent load

The equivalent pressure equation is shown below, indicating the conversion from the pressure peaks on the borehole wall to the vibration circle is (4).

(4) $$P=P_{r}(r_{1}/r_{0}{)}^{-\alpha 1}(r_{2}/r_{1}{)}^{-\alpha 2}$$

$$\alpha_{1}\approx 3\;\mathrm{or}\;=2+\mu /(1+\mu ),\;\alpha_{2}\approx 2-\frac{\mu }{1-\mu }$$

$$r_{1}=(3\sim 5)r_{0};\;\;\;r_{2}=(10\sim 15)r_{0}$$

$$r_{1}=3r_{0},\;r_{2}=10r_{0}$$

*r*_1_—shocking wave radius (m), *r*_2_—cracking area radius (m), *α*_1_, *α*_2_—attenuation index of shock wave and stress wave.

The relative parameters can be calculated according to the characteristics of the blasting simulation, as shown in Table 4. The equivalent pressure on the hypothetical boundary can be approximately calculated by the above calculation process. It also represents the explosive peak value in simulation.

The explosive peak value of the anchorage foundation pit excavation can be further calculated by the above result, and the relative parameters are shown in Table 5. The maximum charge of a single section is 18 kg, and the calculated load is 9.88 Mpa, resulting in an equivalent value Pmax = 9.88 MPa of elastic vibration. In addition, the rise and fall times are 3 ms and 12 ms, respectively, and the total blasting time is 1 s.

#### 3.2.3. Damping Calculation

Damping is a main factor that causes energy consumption and reduces the amplitude of structural vibration. Furthermore, as a critical factor in wave propagation, damping is beneficial to the attenuation of shock wave energy. Moreover, the Rayleigh [27] damping coefficient, a common damping factor in explosive calculations, assumes the damping matrix as a linear system. It is the combination between mass matrix and stiffness matrix, as shown in Equation (5):

(5) [C]=α[M]+β[K]

where damping matrix *C* represents the linear combination between mass matrix *M* and stiffness matrix *K*; *α* is the mass ratio damping coefficient; and *β* is the stiffness ratio damping coefficient.

*α* and *β* are calculated by Equation (6):

(6) $$\alpha =\frac{2\xi }{\omega_{i}+\omega_{j}}\omega_{i}\omega_{j};\;\;\;\beta =\frac{2\xi }{\omega_{i}+\omega_{j}}$$

*ω*_i_ and *ω*_j_ are two separate reference frequencies of stage i and j, *ξ* indicates the structural damping ratio.

The value of *ξ* depends on the structural type, material property, and loading wave (*ξ* is 0.01 due to the lack of damping ratio in the experiment). By applying eigenvalue analysis for the shock wave, two main vibration periods can be obtained: the first main vibration period is 1.06 s; the second main vibration period is 0.87 s.

#### 3.2.4. Boundary Conditions

In dynamic analysis, conventional boundary conditions would cause enormous deviation by reflecting the wave. Therefore, the viscous boundary conditions proposed by Lysmer and Wassis in 1972 [28] were used. The calculation of the soil damping ratio in the x, y and z directions defines the viscous boundaries, as shown in Equations (7) and (8).

*P* wave:

(7) $$C_{p}=p.A\sqrt{\frac{\lambda +2G}{p}}=\gamma .A.\sqrt{\frac{\lambda +2G}{\lambda .9.81}}=C_{p}.A$$

*S* wave:

(8) $$C_{s}=p.A\sqrt{\frac{G}{p}}=\gamma .A.\sqrt{\frac{G}{\lambda .9.81}}=C_{s}.A$$

$$\lambda =\frac{\nu E}{\left(1+v)(1-2\nu \right)}.G=\frac{vE}{2(1+v)}$$

*C_p_* and *C_s_* are two damping values of *P* wave and *S* wave, *ρ*—density, *λ*—volume elasticity coefficient (kN/m^2^), *G*—shear elasticity coefficient (kN/m^2^). *E*—elastic modulus (kPa), *ν*—Poisson ratio, *A*—cross-sectional area.

#### 3.2.5. Simulation on the Blasting Vibration Field

Based on the designed blasting requirements, conventional loose control blasting (φ = 70 mm) method should be applied, with a distance of 50 m from the dangerous rock belt. Working conditions for the simulation are as follows: the horizontal distance between the first-stage slope (close to the side of the slope top) and the unstable rock body is 50 m, which is consistent with the field measurement data. The maximum single-blow blasting charge is 18 kg.

**Figure 8:**
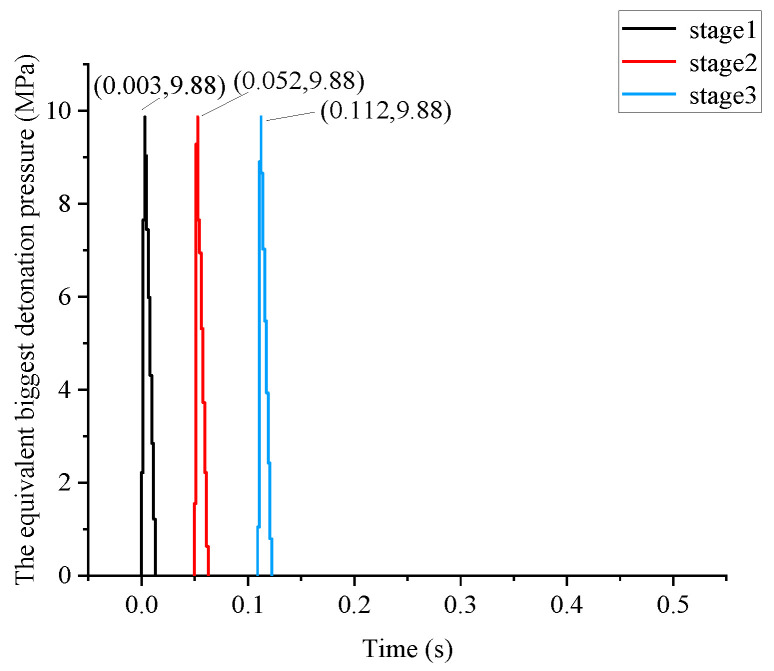
The stress time-history curve of blasting.

**Figure 9:**
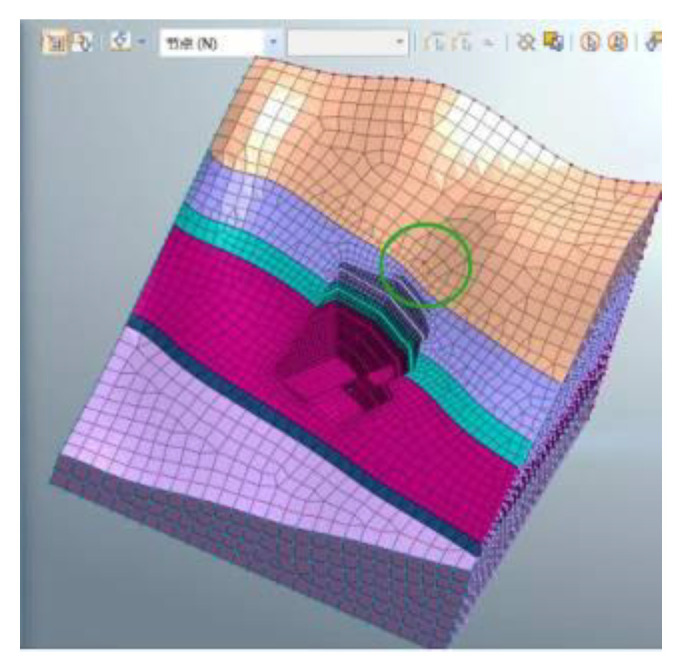
Vibration velocity test point on the foundation pit.

**Figure 10:**
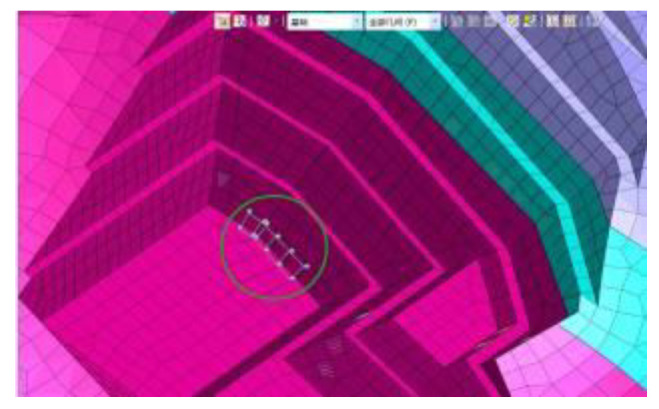
The position and area of loading.

The Mohr–Coulomb elastoplastic material model with a fixed bottom is selected for geotechnical calculation. The surrounding is set as a viscous boundary to deduce the influence of the reflecting wave. The total explosive charge is controlled by stages 1, 3 and 5, with a maximum one-time blasting of 18 kg. As shown in Figure 8, the biggest detonation pressure is 9.88 Mpa; the rising time is 3 ms, the falling time is 12 ms, and the total duration is 1 s. Figure 9 and Figure 10 show the test point on the foundation pit, and the loading area. The blasting load is located in the first stage of the slope (near the side of the slope), while the monitoring point is located at the midpoint of *W*1 and *W*2 on the dangerous rock. Therefore, the calculating process for the horizontal distance between them can be as follows:

(1) Gravity balance and displacement clearing should be carried out before slope excavation;
(2) The static calculation of excavation and support is essential for stages 6, 5, 4, 3 and 2;
(3) Blasting is applied on stages 1, 3 and 5 to retrieve the vibration field.

### 3.3. Analysis of Simulation Results

#### 3.3.1. Blasting Vibration Velocity on the Basement of Perilous Rock

According to Blasting Safety Regulations (GB 6722—2014) [29], the Sadaovsk formula is used to calculate the allowable range of blasting vibration:

(9) $$R={\left(\frac{K}{V}\right)}^{\frac{1}{2}}Q^{\frac{1}{3}}$$

*R*—the allowable safety distance for blasting (m); *V*—the allowable safety vibration velocity for blasting (cm/s); *Q*—blasting charge, total charge of simultaneous blasting (kg); *K,α*—parameters and attenuation coefficients related to terrain and geological conditions between the explosive location and protected object.

The blasting safety procedures take the peak particles vibration velocity and main vibration frequency of the protected object as the blasting vibration criterion, and blasting vibration velocity control is carried out in the area that may affect the surrounding houses. From a safety perspective, the vibration velocity of a dangerous rock burst is controlled within 1.0 cm/s, and the blasting vibration speed of civil buildings should be controlled within 0.5 cm/s, to not affect the surrounding buildings or disturb the people. Therefore, the parameters, such as *K* = 200 and α = 1.8, are determined based on the blasting scheme and experience from the project in the Three Gorges Reservoir area. At the same time, the explosion point is located 50 m outside the unstable rock, the maximum single blasting charge is 18 kg, and the vibration velocity of the dangerous rock base is 1.0 cm/s, according to the Sadaovsk formula.

#### 3.3.2. The Velocity Curve of the Blasting Vibration on the Basement under Perilous Rock

**Figure 11:**
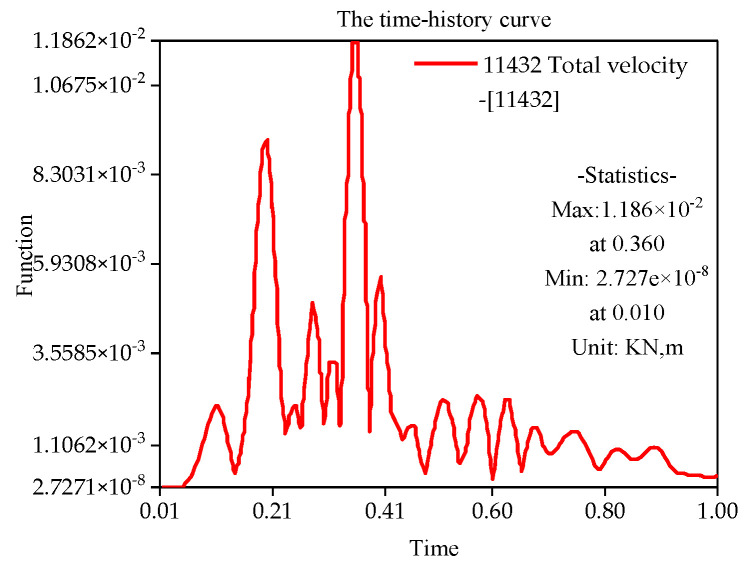
Total vibration curve on the basement of perilous rock.

**Figure 12:**
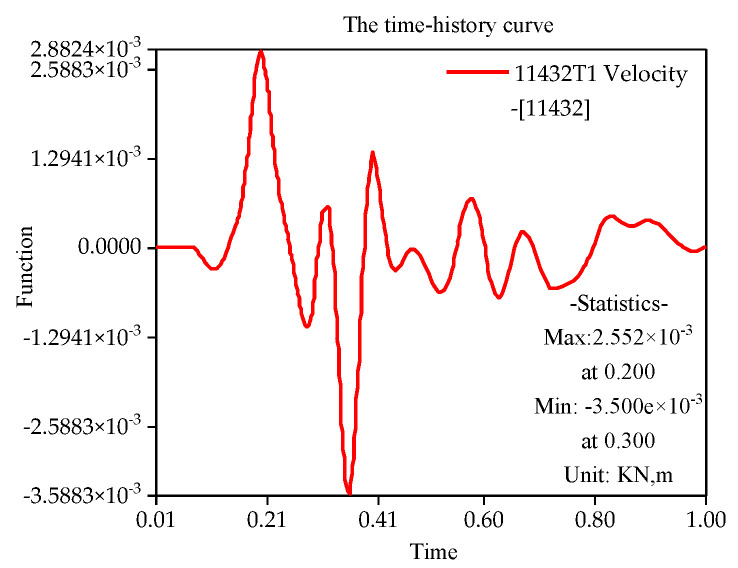
T1 Vibration curve on the basement of perilous rock.

**Figure 13:**
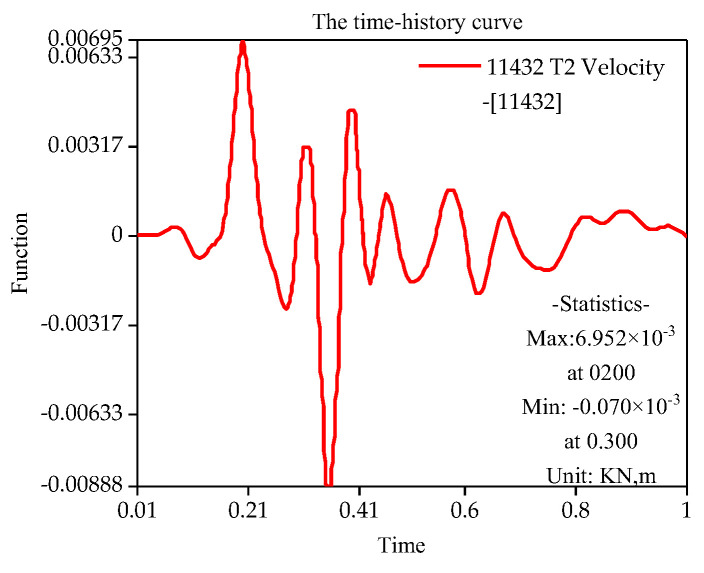
T2 Vibration curve on the basement of perilous rock.

**Figure 14:**
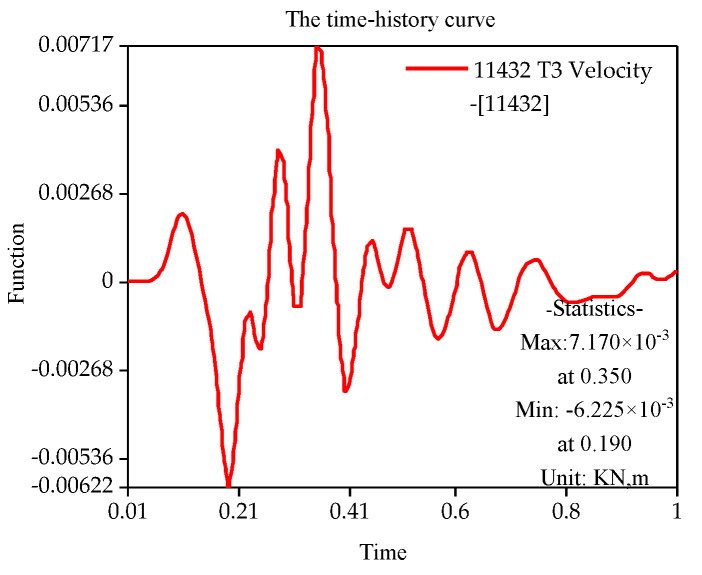
T3 Vibration curve on the basement of perilous rock.

With the finite element simulation, Figure 11, Figure 12, Figure 13 and Figure 14 show the velocity curve of blasting vibration on the basement under the perilous rock.

The location of blasting leading to the movement of the vibration curve on the basement of perilous rock at T1 and T2 is obviously opposite to T3, and the total vibration curve is coupled by T1 to T3. Furthermore, in terms of single velocity, T1 and T2 appear to be the peak; meanwhile, T3 is the minimum within 0.21, and the vibration velocity of T3 attains the maximum of 0.72 cm/s within 0.35 s. The total vibration velocity on the basement of the perilous rock reaches a maximum value of 1.19 cm/s within 0.36 s. The vibration velocity gradually decays after attaining the maximum value.

#### 3.3.3. Blasting Vibration Velocity near the Pit

**Figure 15:**
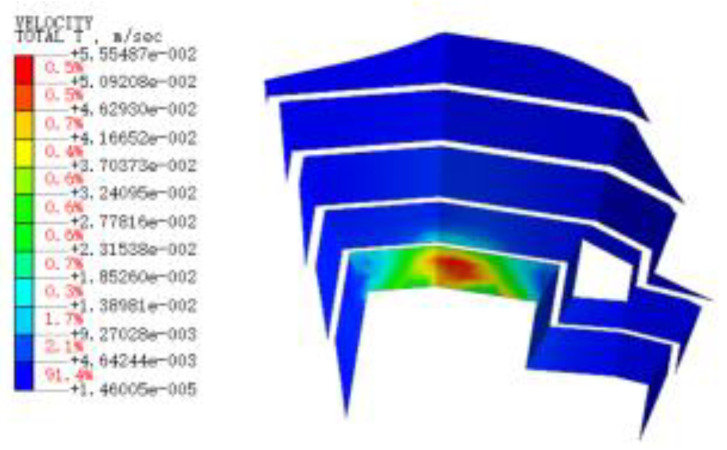
The vibration velocities of retaining walls at 50 ms.

**Figure 16:**
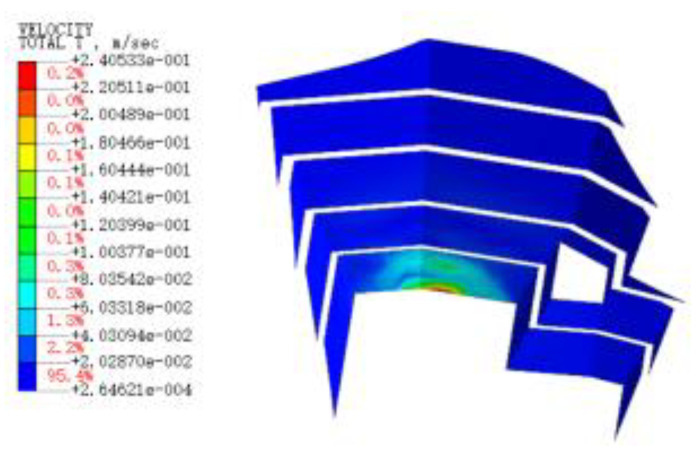
The vibration velocities of retaining walls at 100 ms.

**Figure 17:**
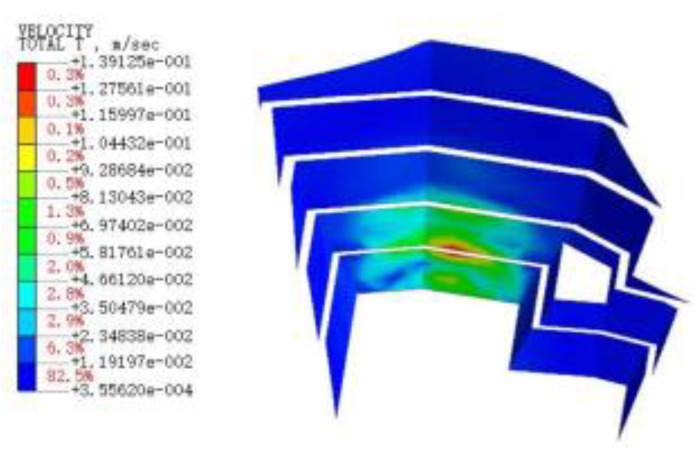
The vibration velocities of retaining walls at 200 ms.

**Figure 18:**
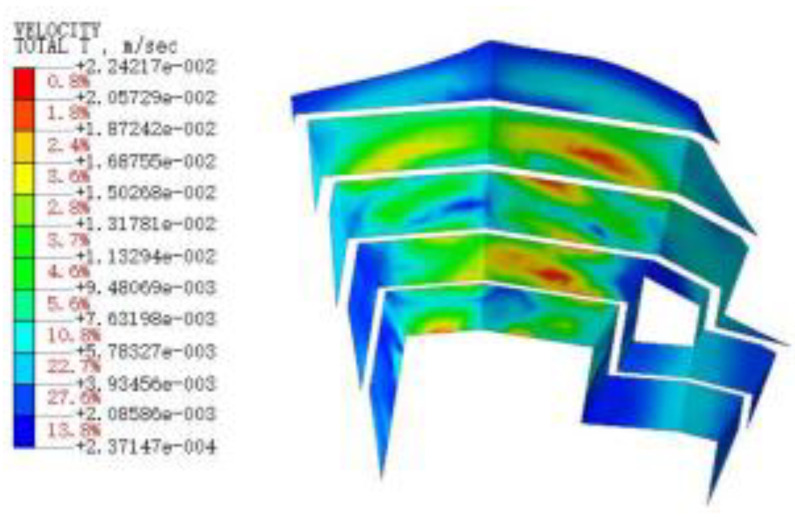
The vibration velocities of retaining walls at 300 ms.

**Figure 19:**
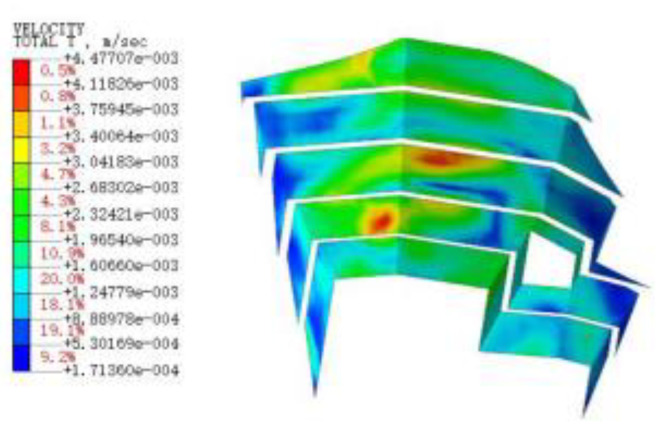
The vibration velocities of retaining walls at 500 ms.

**Figure 20:**
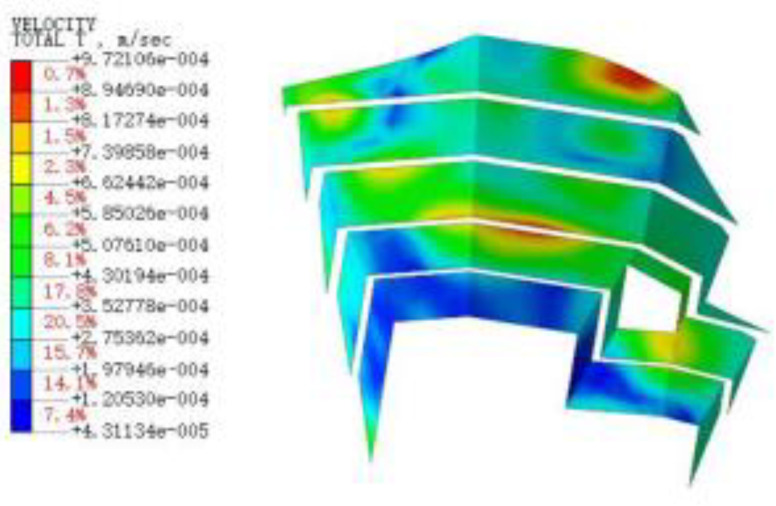
The vibration velocities of retaining walls at 800 ms.

**Figure 21:**
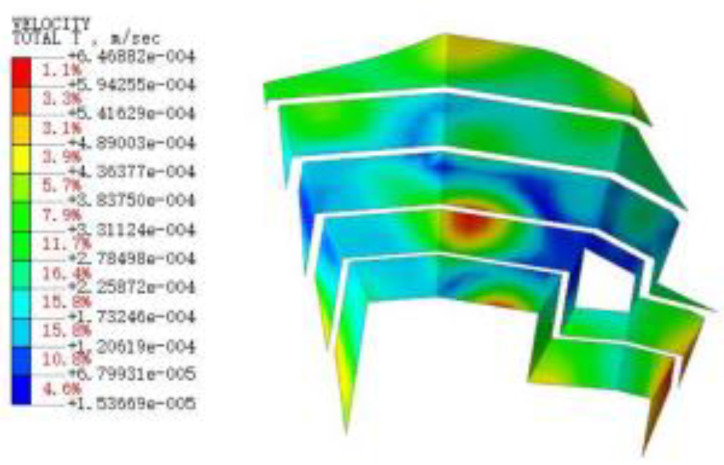
The vibration velocities of retaining walls at 1000 ms.

Figure 15, Figure 16, Figure 17, Figure 18, Figure 19, Figure 20 and Figure 21 show the vibration velocities of the retaining walls at 50 ms, 100 ms, 200 ms, 300 ms, 500 ms, 800 ms, and 1000 ms throughout the blasting simulation. The blasting vibration mainly impacts the first-stage slope at 50 ms. The vibration velocities attain the largest value of 24.05 cm/s on the first-stage slope at 100 ms. This significantly impacts the edge of the retaining walls of the second-stage slope. Moreover, the blasting vibration has an influence on all retaining walls after 300 ms. The blasting load exhibits a rising tendency at first, and then decreases, with the values of the vibration velocities ranging from 50 ms to 1000 ms. The variation trend for the vibration velocity and load is also similar, but slightly lagged compared to the blasting load.

#### 3.3.4. Stability Analysis of the Slope

According to the geological prospecting reports, the ground surface bedrock interface of the higher bank mainly consists of mudstone. It is a relatively water-resistant stratum, with a steep slope of sandstone. Therefore, the bedrock fissure water is comparatively poor, and the influence of rainfall is temporarily ignored. The nonlinear time history +SRM method in the model was used to calculate the slope under complex terrain and geological conditions, which has the important advantage of considering the soil constitutive relationship and the deformation influence on stress. Furthermore, the worst blasting condition on the first-stage slope (near the side of the slope) is simulated, with a horizontal distance of 50 m away from the perilous rock, and the maximum single-blow blasting charge of 18 kg.

**Figure 22:**
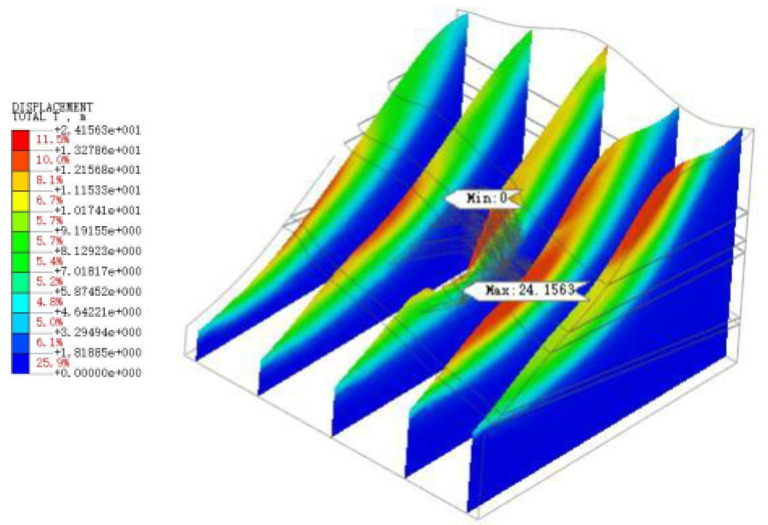
Overall displacement of slope.

**Figure 23:**
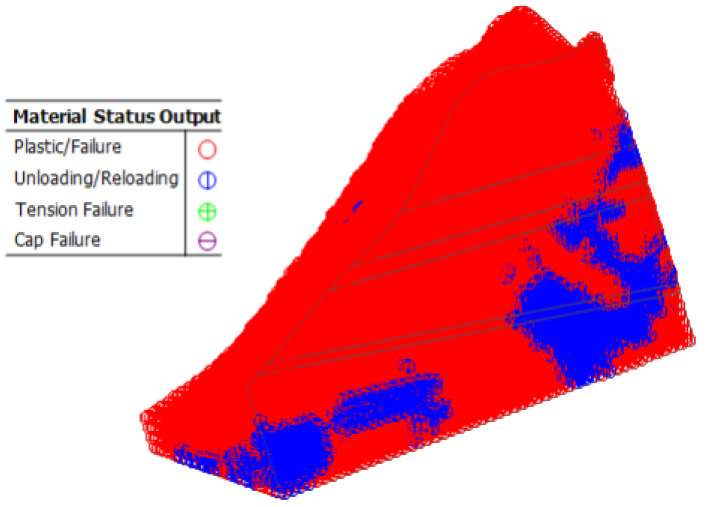
Plastic zone range of slope.

**Figure 24:**
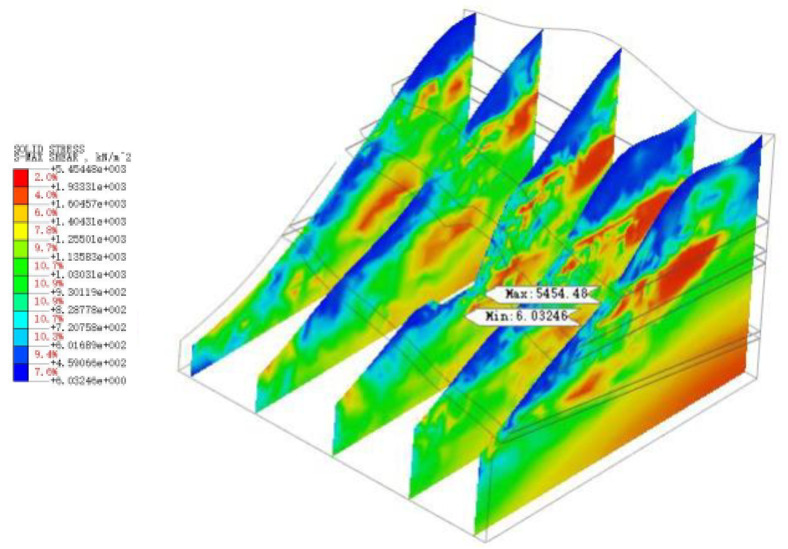
The maximum shear stress of slope.

**Figure 25:**
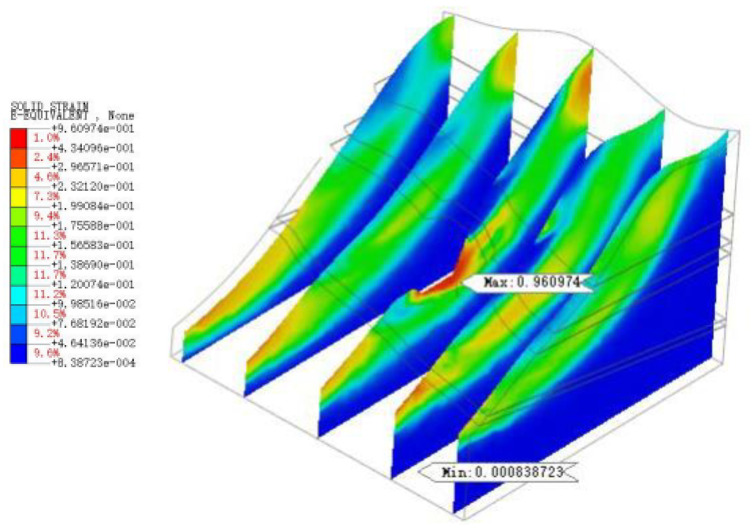
Equivalent strain of slope.

**Figure 26:**
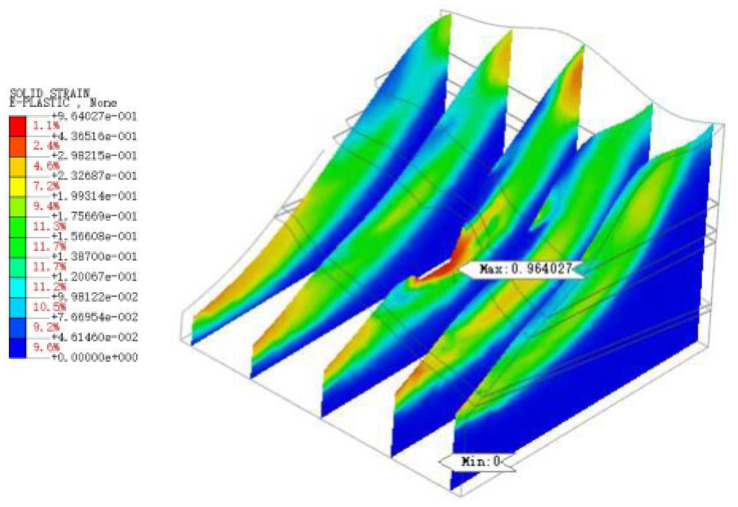
Equivalent strain of slope.

As shown in Figure 22, Figure 23, Figure 24, Figure 25 and Figure 26, the potential slip surface of the anchorage foundation pit is judged by the metrics, including overall displacement, slope plastic zones, effective plastic strain and equivalent strain. In addition, the potential slip surface on the rock mass behind the pit is sheared from the foundation pit of the slope foot. The results show that the overall instability of the slope will occur in the weak zone and the stress concentration area (plastic zone), and its soil element will undergo permanent deformation in varying degrees. When the above parts are connected with each other, the overall instability of the slope will appear in an interconnected shear fracture surface.

Based on the strength reduction method, the 2D dynamic safety factor of the slope is 1.38, and the 3D value is 1.78, as shown in Table 6.

## 4. Experiment on Blasting Vibration

The finite element model data based on risk control measures indicate that the vibration in the unstable rock belt is comparatively small, and slope stability is good. According to the results, construction safety can be ensured. Therefore, the above measures were applied in the actual project, and the field data was monitored in real time.

### 4.1. Experiment Scheme for Blasting Vibration

#### 4.1.1. Testing Devices

**Figure 27:**
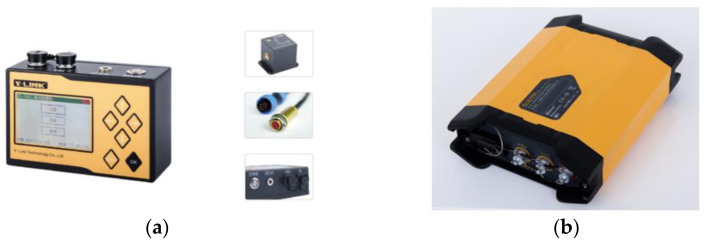
Testing devices: (**a**) Vibration monitor; (**b**) Slope deformation monitor.

Figure 27 shows the two testing devices for the blasting vibration monitoring experiment. The vibration monitoring test must satisfy professional standards and regulations, such as the *safety regulations for blasting* and *Code for measurement methods of dynamic properties of subsoil*. The GNSS slope deformation monitor station is used in monitoring slope deformation, and the GNSS relay station is installed close to the site. Therefore, it is convenient to transmit signals to a central system, which can achieve persistent and unattended data monitoring.

#### 4.1.2. Measuring Point Arrangement

(1) Blasting vibration monitoring point

**Figure 28:**
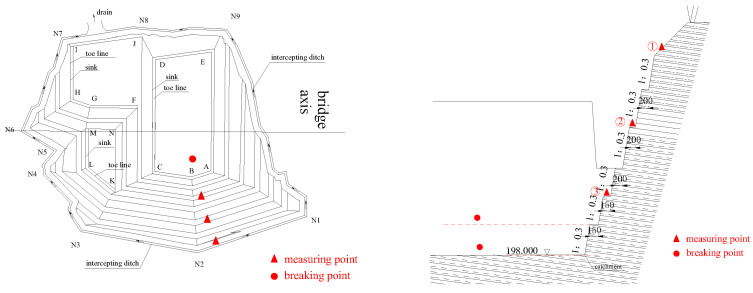
Location of blasting vibration monitoring point.

**Figure 29:**
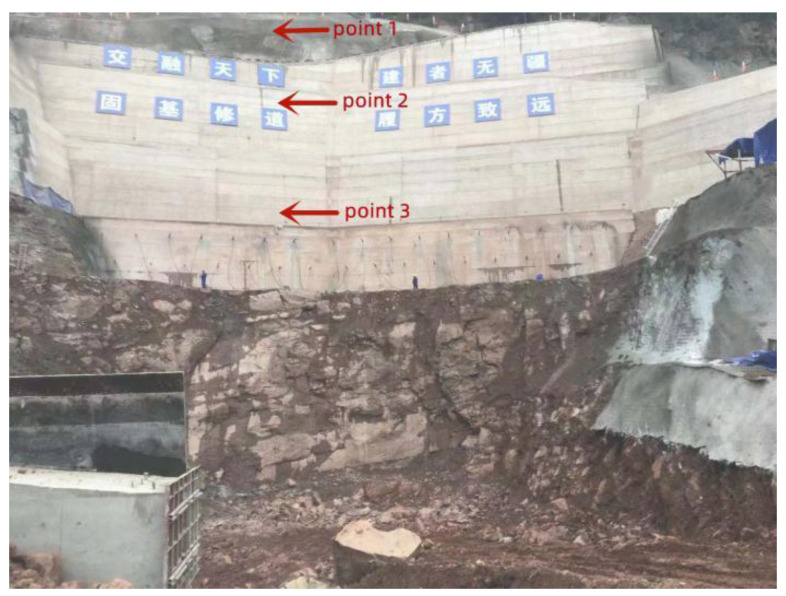
Construction layout of the blasting vibration monitoring points.

In the blasting experiment, three test lines were arranged with three measuring points spaced on the steps, as shown in Figure 28 and Figure 29. The distance between test points and blasting sources is shown in Table 7, and the overall number of blasting times is 6, of which 5 are for the penultimate level of the foundation pit, and 1 time for the lowest level.

(2) Slope deformation monitoring point

**Figure 30:**
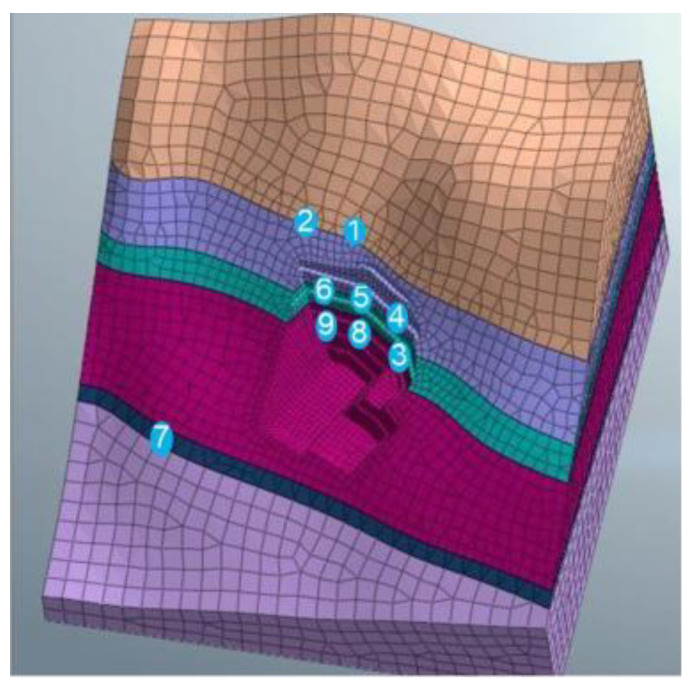
The monitoring point arrangement of slope deformation.

**Figure 31:**
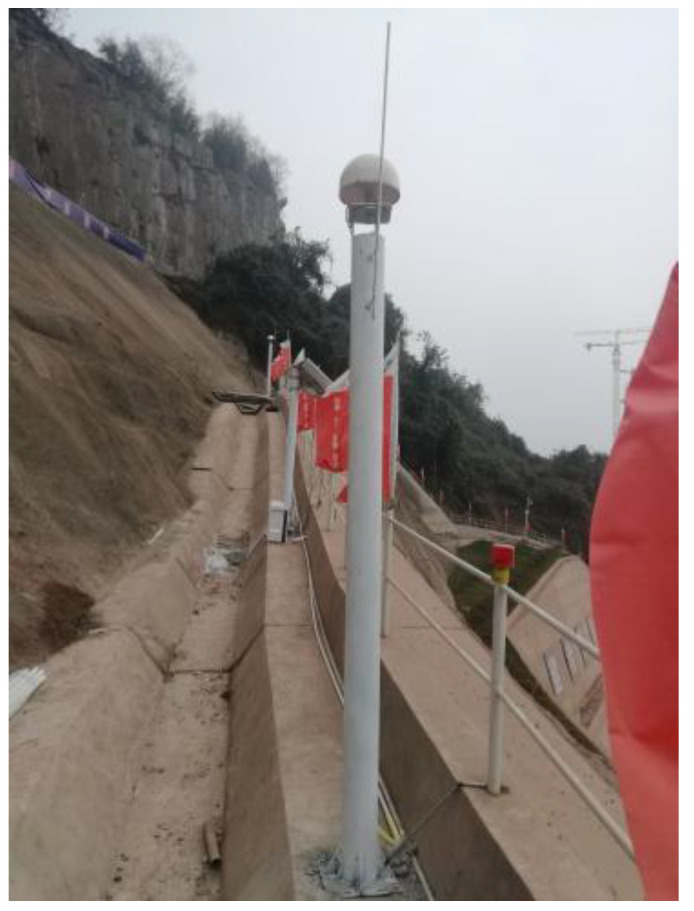
The displacement monitoring device of construction site.

The slope deformation monitoring points were arranged on all levels of the slope platforms, and the distance between each point varied from 15 m to 30 m. According to the observation marks on the stable stones, the basic monitor point was arranged in a stable area, far away from the monitoring slope. In addition, 9 surface horizontal displacement monitoring points were set for the slope. According to Blasting Safety Regulations (GB 6722-2014) [29], the sampling frequency used to measure the vibration in the field test was 50 Hz, and referring to the allowable range of blasting vibration calculated by the Sadaovsk formula, the maximum change ratio of horizontal displacement on the slope deformation monitoring point was 10 mm/d, and the cumulative amount of change warning value is 50 mm. According to the evaluation method of monitoring and alert value stipulated in “Technical Standard for Monitoring Construction Foundation Pit Engineering” (GB50497-2019) (pp. 33–35) [30]; also combined with the characteristics of this foundation pit excavation project, the displacement monitoring, and the alert value range is 40–50 mm. However, the displacement alert value of the current project is the maximum accumulated change of horizontal displacement at the monitoring point of the foundation pit slope. Therefore, the final displacement alert value is 50 mm, and the alert value of the maximum displacement change rate is 10 mm/d. The arrangement of the test points is shown in Figure 30, and the construction site is shown in Figure 31.

### 4.2. Blasting Vibration Test Results

#### 4.2.1. Monitoring Data of Blasting Vibration Velocity

In total, the blasting experiment monitored 17 groups of effective data that contain vibration amplitude and dominant frequency from three directions. The first blasting experiment comprised four blasting times within two days and collected five sets of effective monitoring data on the penultimate level of the foundation pit. The second test obtained a set of valid monitoring data on the lowest level within another two days. The third explosive work using a blasting charge of 352 kg was completed in one day and obtained all the data for points 2 and 3, as shown in Table 8. During the test, the measured vibration was filtered to eliminate the obvious interference vibration (more than three times the designed vibration speed, or less than two times the average minimum vibration speed).

#### 4.2.2. Monitoring Data of Slope Displacement

**Figure 32:**
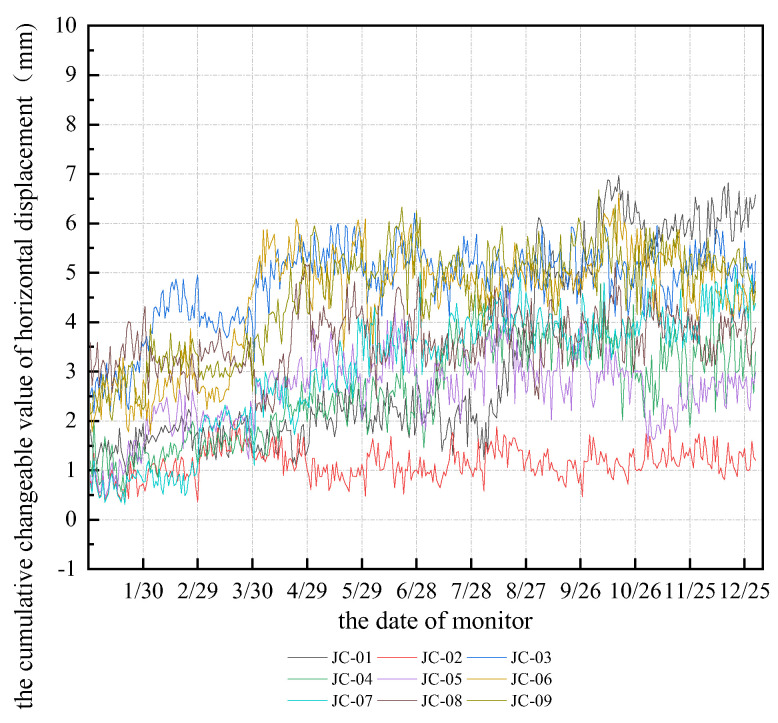
The cumulative changeable curve on annual slope surface horizontal displacement.

Horizontal slope displacement monitoring on the surface is the main task of the project, which can last for one year. The monitoring data show that the displacement of the slope surface had a steady growth trend, and it increased with the foundation pit slope excavation depth. Furthermore, the peak of the horizontal displacement in monitoring point 4 was approximately 12 mm, which was 24% of the control value. Nevertheless, the cumulant displacement index was less than the alert value, and the maximum ratio of displacement change was about 4 mm/d, which was less than the alert value of 10 *mm/d*. Moreover, the monitor data for other points were far lower than the alert value, which meets the requirements of the designed construction. The horizontal displacement monitoring curve is shown in Figure 32.

Horizontal slope displacement monitoring is the main work, which lasts for one year. The monitoring data show that the slope displacement increases with the increase of depth, but the increasing trend is stable. In addition, the peak horizontal displacement of monitoring point 4 was about 12 mm, and when the cumulant displacement index was less than the alarm value, it reached 24% of the control value, and the maximum ratio of displacement change was about 4 mm/d, less than the alarm value 10 mm/d. The monitoring data of other points are much lower than the warning value, which meets the requirements of design and construction. The horizontal displacement monitoring curves are shown in Figure 32.

### 4.3. Comparison between Experiment and Simulation

The data of the finite element simulation are shown in Section 3.3, and the data of the field measurements are shown in Section 4.2, see Table 9. In the field measurement data, the maximum vibration velocity of the dangerous rock foundation is 54.45% of that of the finite element simulation, while at the location of the foundation pit retaining structure, the ratio value is 74.25%. The results of the finite element simulation show that the construction data can ensure the construction safety of the site. However, the data obtained from the simulation has higher values than the field measurement data. The reason is that not all conditions can be fully simulated, thus the most unfavorable conditions are considered, and the numerical calculation is more conservative. In conclusion, the finite element simulation in this paper is effective and is useful to the actual engineering project.

## 5. Conclusions

This paper aims at the challenges of the foundation pit excavation of a suspension bridge project in the Three Gorges Reservoir area. A series of comprehensive risk control measures have been put forward by applying a hierarchical excavation and minimum charge blasting method. Meanwhile, the feasibility of the scheme is also verified via a finite element simulation, and further applied to the actual construction. There are five main conclusions as follows:

(1) The layered and graded excavation scheme can effectively reduce slope deformation, which is critical for construction safety. Therefore, excavation should follow the construction sequence, and the minimum explosive blasting method should be adopted in the excavation of the foundation pit. Thus, construction safety could be ensured below the dangerous rock belt.
(2) The blasting is located on the first slope, close to the side of the slope top, with an approximate horizontal distance of 50 m from the blasting location to the unstable rock mass. The maximum single-blow blasting charge is 18 kg. Meanwhile, the nonlinear time-history analysis method is used to simulate the vibration field of the anchorage foundation pit slope. As a result, the maximum value of the total velocity can reach 1.19 cm/s within 0.36 s, and the vibration velocity gradually decays after attaining the maximum value.
(3) Blasting of first-stage slope has a significant influence on the edge retaining walls of second-stage slope, and the vibration velocities attain 24.05 cm/s in 100 ms. Furthermore, the protecting wall should be comprehensively determined by *safety regulations for blasting* (concrete), numerical simulation results, and field measurement data.
(4) The blasting is located on the first slope (close to the side of the slope top), with a maximum single blasting charge of 18 kg. The two-dimensional dynamic safety factor of slope is 1.38, while the three-dimensional factor is 1.78 based on the strength reduction method. Both safety factors of the excavation slope meet the design requirements of the blasting excavation process.
(5) The foundation pit project of the suspension bridge mentioned in this paper has been successfully completed. The results show that risk control measures can guarantee engineering qualities and ensure construction safety. The engineering experience can be set as a reference for similar projects around the world.

## Figures and Tables

**Table 1:** Physical and mechanical parameters of the main rock and soil layers.

Strata	Natural Bulk Density kN/m^3^	Saturated Compressive Strength (MPa)	Shear Strength C (kPa)	Shear Angle Φ (°)	Poisson Ratio μ	Elastic Modulus Ee (GPa)
Mudstone	25.4	5.65	430	30.23	0.32	1
Sandstone	25.1	24.4	1720	34.9	0.25	6.7
Sandy mudstone	25.4	11.2	830	32.39	0.31	2.3

**Table 2:** The design parameters of the supporting structure.

Material Type	Model Element	Elastic Modulus (Mpa)	Poisson Ratio	Density (g.cm^−3^)	Size/m
Anchor bar	implantable truss element	200,000	0.2	7.85	4, 6, 9 m
Anchor cable	implantable truss element	200,000	0.2	7.85	Length 40 m, grouting Length 9 m
Protecting walls	plan element	30,000	0.3	2.5	Thickness 0.5 m

**Table 3:** Performance index of 2 rock emulsion explosives.

Name	Performance Index				
Number 2 rock emulsion explosion	Transmission distance (cm)	Brisance (mm)	Detonation velocity (m/s)	Work capacity (ml)	Density (g/cm^3^)
	≥3	≥12	≥3200	≥260	0.95–1.30

**Table 4:** Blasting parameters.

Parameter	Value
The initial pressure peaks of shock wave *Pr* (pa)	760,000,000
Density of rocks *ρ_r_* (kg/m^3^)	2300
Compression wave velocity of rock body *Cer* (m/s)	4000
Explosive density *ρ*_0_ (kg/m^3^)	1100
Explosive velocity *D* (m/s)	1200
Explosive force *P_e_* (pa)	570,000,000
Equivalent pressure *P* (pa)	9,878,362
Shock wave radius *R*_1_ (m) (3 times the hole diameter)	0.105
Explosive radius (m)	0.0275
Borehole radius *RO* (m)	0.035
Crack area radius *R*_2_ (m) (10 times the hole diameter)	0.35
*α*_1_	2.230769
*α*_2_	1.571429
Poisson ratio	0.3

**Table 5:** Blasting parameter calculation.

Initiation Position	The Maximum Charge of Section/kg	The Maximum Explosive Pressure/Mpa	Rise Time/ms	Fall Time/ms	Total Duration/s
Bench slope	18	9.88	3	12	1

**Table 6:** Safety factor of slope stability on anchorage foundation pit.

Calculation Condition	Two-Dimension Overall Stability	Three-Dimension Overall Stability	Normal Safety Factor
Foundation pit dynamic excavation	1.38	1.78	1.25

**Table 7:** The distance between test points and blasting sources.

Blasting Location	Test Point	Elevation *H/*m	Horizontal Distance *D/*m	Explosive Source Distance *R*/m	Sampling Frequency Hz
Penultimate level of foundation pit	Point 1	8	15.15	17.13	50
	Point 2	24	23.70	33.73	50
	Point 3	40	32.50	51.54	50
The lowest level of foundation pit	Point 1	16	19.05	24.88	50
	Point 2	32	27.60	42.26	50
	Point 3	48	36.40	60.24	50

**Table 8:** Monitoring data of blasting vibration.

Number	Monitor Point	Total Blasting Charges/kg	Dominant Frequency/Hz			The Peak of Vibration Velocity/cm/s		
			Radial	Tangential	Vertical	Radial	Tangential	Vertical
1	Point 2	352	45	20	16	0.795	1.748	0.712
2	Point 3		11	15	16	0.647	0.648	0.552
3	Point 1	564	15	16	21	17.857	0.009	0.008
4	Point 2		39	26	21	1.355	0.626	3.155
5	Point 3		38	56	37	2.325	0.526	3.123
6	Point 1	413.2	23	24	24	2.831	0.002	0.002
7	Point 2		23	22	21	1.392	1.342	1.667
8	Point 3		24	56	24	1.925	1.302	2.861
9	Point 1	384	24	24	24	2.946	0.002	0.001
10	Point 2		24	23	15	0.553	0.460	0.742
11	Point 3		23	56	23	0.856	0.454	1.302
12	Point 1	417	25	26	26	2.268	0.002	0.001
13	Point 2		25	27	19	0.389	0.215	0.325
14	Point 3		24	24	23	0.493	0.225	0.554
15	Point 1	493	37	38	38	14.351	17.554	16.092
16	Point 2		37	28	26	2.608	0.935	3.638
17	Point 3		18	26	19	1.340	2.657	1.449

**Table 9:** Comparison between experiment and simulation.

Location	Maximum Vibration Velocity (FEM)	Maximum VIBRATION velocity (EX)
Base of dangerous rock	1.19 cm/s	0.648 cm/s
Foundation pit retaining structure	24.05 cm/s	17.857 cm/s

## Data Availability

Not applicable.

## Nomenclature

*Q_max_* (kg)	the total blasting charge
*L*2 (m)	the total length
*Ρ* (kg/m^3^)	the blasting density
*P*_0_ (kg/m^3^)	explosive density
*D*_0_ (m/s)	explosive detonation velocity
*d_b_* (m)	explosive diameter
*d_c_* (m)	borehole diameter
*P_r_* (Pa)	the initial pressure peaks of the shock wave in the rock body
*ρ_r_* (kg/m^3^)	density of the rock body
*C_er_* (m/s)	compression wave velocity of the rock body
*r*_1_ (m)	shock wave radius
*r*_2_ (m)	cracking area radius
*α*_1_, *α*_2_	attenuation index of the shock wave and stress wave
*C*	damping matrix
*M*	mass matrix
*K*	stiffness matrix
*β*	the stiffness ratio damping coefficient
*ω_i_* and *ω_j_*	two separate reference frequencies of stage *i* and *j*
*ξ*	the structural damping ratio
*C_p_* and *C_s_*	two damping of *P* wave and *S* wave
*ρ*	density
*λ* (kN/m^2^)	volume elasticity coefficient
*G* (kN/m^2^)	shear elasticity coefficient
*E* (kPa)	elastic modulus
*ν*	Poisson ratio
*A* (m^2^)	cross-sectional area
*R* (m)	the allowable safety distance for blasting
*V* (cm/s)	the allowable safety vibration velocity for blasting
*Q* (kg)	blasting charge, total charge of simultaneous blasting
*K*, *α*	parameters and attenuation coefficients related to terrain and geological conditions between the explosive location and protected object
